# ‘A confession of ignorance’: deaths from old age and deciphering cause-of-death statistics in Scotland, 1855–1949

**DOI:** 10.1080/1081602X.2014.1001768

**Published:** 2015-02-05

**Authors:** Alice Reid, Eilidh Garrett, Chris Dibben, Lee Williamson

**Affiliations:** ^a^ Cambridge Group for the History of Population and Social Structure, Department of Geography, University of Cambridge, Cambridge, UK; ^b^ Department of Geography and Sustainable Development, University of St Andrews, St Andrews, UK; ^c^ Research Institute of Geography and the Lived Environment, School of GeoSciences, University of Edinburgh, UK

**Keywords:** cause of death, mortality, Scotland, old age, epidemiological transition

## Abstract

A large amount of the research undertaken in an attempt to discover the reasons underlying the late nineteenth- and early twentieth-century mortality decline in Britain has relied on the statistics published by the Registrars General. The processes by which individual causes of death are recorded and then processed in order to create the statistics are not, however, well understood. In this article, the authors build on previous work to piece together a time series of causes of death for Scotland, which removes many of the discontinuities encountered in the published statistics that result from the Registrar General deciding to update the nosology, or classification system, which was being used to compile his figures. Having regrouped individual causes of death to ‘smooth’ the time series, the authors use the new groups to examine the changing causes of death in Scotland for selected age groups, before turning to undertake a detailed examination of mortality amongst those aged 55 or more. The authors find that when deaths from ‘old age’ in the latter age group are separated from other ‘ill-defined’ causes, it becomes obvious that there was a ‘rebranding’ of cause of death. The authors then use individual-level data from two Scottish communities to further dissect the roles played by ‘informants’ and ‘doctors’ in this rebranding, in order to see how these roles may have altered over time and what the consequences might be for one's view of how mortality changed in Scotland between 1855 and 1949. Finally, the authors argue that their findings have important implications for some of historical demography's most prominent theories: the McKeown thesis and the theory of epidemiological transition.

## 1. Background

Most major theories proposed to describe or explain the secular decline in mortality from the mid nineteenth to the late twentieth century in Europe, the USA and other more economically developed countries have been based on time series of the numbers of people dying from particular diseases or conditions. Changes in the risks of death from different diseases as mortality declined form the basis of Omran's (1971) theory of epidemiological transition. According to this theory, mortality before the decline was characterised by an age of pestilence and famine. This was followed by an age of receding pandemics during the decline, and an age of degenerative and man-made diseases in the post-decline era. An age of delayed degenerative diseases has been added by subsequent authors as a fourth stage succeeding Omran's original three (Olshansky & Ault, 1986). Omran's (1971) theory and description of the epidemiological transition were highly dependent on changes in the causes of death over time: in the age of pestilence and famine, cardiovascular disease and cancer were responsible for less than 6% of deaths (p. 517), and the transition is typically characterised by a ‘shift from infectious to degenerative disease’ (p. 518). Omran drew on published cause-of-death statistics from selected countries, chosen to represent the spectrum of health experience across the globe; they included England and Wales, Sweden, Japan, Chile and Sri Lanka (then Ceylon), and he later developed his ideas with data from the USA (Omran, 1971, 1977).

Thomas McKeown (1976a) also used cause-of-death data for England and Wales as the basis for his controversial theory regarding the reasons for the country's mortality decline, which stated that the primary reason for the decline was improving nutrition, which, he argued, was due to the increased standards of living being enjoyed by the population.^1^ He drew this conclusion because he maintained that nothing else could have affected the category of airborne diseases – dominated by tuberculosis, but also containing pneumonia and bronchitis – which was responsible for the largest percentage declines in mortality. McKeown's thesis has been widely debated, with criticism being made of his calculations and his concentration on tuberculosis while ignoring bronchitis and pneumonia, and particularly his narrow conceptualisations of public health and medical intervention (Fairchild & Oppenheimer, 1998; Razzell, 1965; Szreter, 1988).^2^ Nonetheless, McKeown's idea that health differentials are strongly based on material living standards retains currency.

Both McKeown and Omran appear to have assumed that the official statistics of cause of death could be straightforwardly interpreted at face value. However, there is a substantial and growing body of work relating to the problems of interpreting cause-of-death statistics, and here we wish to demonstrate some of the issues and their implications by using Scotland as an example. Before proceeding further, however, it is worth sketching out the process whereby the circumstances of a multitude of individual deaths are transformed into official statistics in Great Britain.

Civil registration – the secular and state-run registration of births, deaths and marriages – began in England and Wales in 1837, and in Scotland in 1855. When an individual died, that death had to be reported to a local registrar by an informant, who was usually a relative or neighbour of the deceased, and who had to provide not only the date of the death, but certain details of the deceased, including their age, sex and cause of death. The registration was supposed to be accompanied by a certificate from a doctor who had recently attended the deceased, certifying the cause of death. In Scotland, the doctor delivered the certificate straight to the registrar, but in England and Wales the doctor handed the certificate to the informant, who took it to present to the registrar when registering the death.^3^ A small minority of deaths – mainly those from violence or occurring in suspicious circumstances – were referred to the local coroner (if in England and Wales) or procurator fiscal (if in Scotland), who would undertake an enquiry into the cause of death. Autopsies, however, were rarely performed, even in these cases. In Scotland, the local registers were kept in duplicate, with one of the copies being forwarded to the office of the Registrar General (Sinclair, 2000, p. 36). In England, the registrars made certified copies of the contents of their registers to be sent to the superintendant registrar, who then sent them on to the Registrar General (British Parliamentary Papers, 1836). Once received, the clerks at the offices of the Registrars General tallied the numbers of deaths by different causes, ages and sexes, and the resulting tables were published in the annual and decennial reports of the Registrar General for England and Wales and the Registrar General for Scotland.

There are many stages throughout the process of the compilation of mortality statistics which might prompt us to be uncertain about just how much reliance can be placed on the cause of death. Not all deaths were certified by a doctor, and the causes offered by relatives and neighbours tended to be far less scientific and almost certainly less accurate than those offered by a trained medical practitioner. Even those deaths where the cause was certified by a doctor might be unreliable. The doctor might not have seen the deceased in their last illness and, in coming to their diagnosis, might themselves be relying on the relatives' reports of symptoms. Without an autopsy or medical tests it would have been difficult to diagnose an underlying cause of death with the level of accuracy that we, perhaps mistakenly, take for granted today. Many of the proffered causes were symptomatic in nature, particularly in the early part of the civil registration period, and many symptoms could be indicative of a number of different underlying conditions. There is also a suspicion, particularly in England and Wales where the doctor's certificate was handed to the relative to take to the registrar, that doctors might have avoided certain causes of death either to save the relatives from pain or embarrassment – if, for instance, the deceased had succumbed to a venereal disease – or to save themselves from seeming incompetent, as might be the case if a patient under their care had died as a result of puerperal fever. The relative effects of the various influences on the reliability of causes of death offered to the registrars will have changed over time as both medical provision and certification increased. To further complicate matters, as medical knowledge advanced, new theories of disease causation were introduced, and there was a move away from the description of symptoms to the identification of the underlying aetiology. These and other issues relating to what is recorded as the cause of death have been discussed by a number of scholars (including Alter & Carmichael, 1996, 1998, 1999; Anderton & Leonard, 2004; Hardy, 1994; Kippen, 2011; Kunitz, 1999; Reid & Garrett, 2012; Risse, 1997; Williams, 1996).

A related, but slightly different, set of factors affects the process by which each cause of death as written on the registration certificate was translated into a published statistic. Working backwards from the published figures, one of the main issues in comparing causes of death over time is the change in the nosologies – the lists of causes of death used to categorise deaths. In 1855, the Registrar General for Scotland was using a nosology based on 109 causes, but, by 1949, 255 causes were being used, and the latter were not simply subdivisions of the former; as medical knowledge developed and theories concerning the causes of disease changed, so did the way the recorded causes were grouped together and, over time, some causes previously thought to be the same were separated out. One such case was that of typhus and typhoid, which, until 1865 in Scotland and 1869 in England and Wales, were placed in the same category because their very different aetiology had not been realised. Various causes of death were frequently transferred between different categories as nosologies changed, and the ‘unknown and ill-defined’ category shrank over time as the identification of causes of death improved. The problems of trying to follow individual causes of death, or groups of causes, over time have been addressed by a number of authors (including Janssen & Kunst, 2004; Kippen, 2011; Kunitz, 1999; Meslé & Vallin, 1996; Wolleswinkel-Van den Bosch, Van Poppel, & Mackenbach, 1996). Some of those wanting to examine mortality over time have eschewed the use of cause-of-death statistics entirely due to these problems (for example, Lee, 1991), and others have restricted their use of national statistics to one side of a major nosological change (Woods & Shelton, 1997). Others have restricted their attention to certain, more easily identifiable diseases, which, they argue, are less likely to have changed over time (Alter & Carmichael, 1996; Woods & Shelton, 1997).

What has been less discussed, although not totally ignored, is the intermediate process of transferring a cause of death on a certificate to the current nosology: in other words, how did the clerks in the General Register Offices decide on the category in which to place a death? It might be thought that this was straightforward, but those providing information on the cause of death, whether they were doctors or lay informants, did not always make it easy, using ambiguous or ill-defined terms or offering a selection of different causes. Clerks were almost certainly given instructions on how to deal with tricky cases, but those from the nineteenth century do not appear to have survived.

This article addresses some of these issues related to the recording, classification and interpretation of causes of death for Scotland over the course of the major secular decline in mortality from 1855 to 1949. As indicated above, our aim is to offer further comments on the reliability of official statistics of the cause of death and the implications for the ‘grand theories’ which rely on them.

## 2. Data

This article uses two sources of data, the first being the ‘official’ cause-of-death series published in the annual reports of the Registrar General for Scotland from 1855 to 1949 (Davenport, 2012). We mainly present the data in the form of mortality rates, rather than absolute numbers, and the population data which form the denominators of these rates are taken from the Human Mortality Database (www.mortality.org) and originate from the Registrar General for Scotland's estimates derived from the censuses of Scotland. In order to supplement these data, and to provide detail of the way in which the registered causes of death were translated into official statistics, we use as our second source a series of death registers for the lowland town of Kilmarnock and the highland island of Skye. These data comprise all the individual registrations of death, and include the age, sex, cause of death and whether the death was medically certified, as well as all the other pieces of information required for each deceased person. The time period for which these records are available (1861–1901) is shorter than the period covered by the aggregate data, but this is the period of major mortality decline, at least for adults and children above the age of infancy, and is also a time when certification was increasing and major developments in the knowledge of disease causation were occurring. There were 23,715 deaths in Kilmarnock and 12,285 on Skye. The Registrar General provided annual series of numbers of deaths by cause for the large towns of Scotland and so, because Kilmarnock was one of these large towns, we can compare the causes given at the registration of individual deaths with the distribution of cause of death provided by the Registrar General in order to help us investigate coding and classification issues. Unfortunately, this is not possible for the Isle of Skye because figures for small towns and rural areas were only provided for county aggregates, so Skye was subsumed into a table which combines the statistics for all the insular/rural districts of Inverness-shire together.

As mentioned above, there has been a considerable amount of work published on the problems of reconciling changing nosologies to enable a comparison of causes of death over time (Janssen & Kunst, 2004; Meslé & Vallin, 1996; Wolleswinkel-Van den Bosch et al., 1996). It is difficult to apply nineteenth-century nosologies to twentieth-century data, and vice versa, and studies which create consistent categories over time by following individual causes, and aggregating up when there is an absence of a straightforward transfer from one category to another, are useful over relatively short time frames during which few or minor nosological changes occur. However, over the longer time scale and across major changes, such studies end up with large proportions of deaths in ‘nosologically not meaningful’ categories (Wolleswinkel-Van den Bosch et al., 1996). Some studies redistribute causes rather than aggregating up (Meslé & Vallin, 1996), but this, too, can be problematic, as it is necessary to assume a constant distribution of underlying causes in ill-defined categories, and this seems dangerous when trying to compare causes over time (Janssen & Kunst, 2004, p. 911).

In our approach, we have tried to maintain useful groupings, including ill-defined categories, over a long time period, which we hope will eventually extend up to the late twentieth century. Much of what we have done follows the work of Kippen (2011), who was working on data from Tasmania, although the categories we end up with differ somewhat from hers. We allocated each individual cause listed in the Registrar General's reports to one of the 22 broad ‘chapters’, or classes of cause, of the International Classification of Diseases, version 10 (ICD-10), as if they had been diagnosed today. For example, we assigned ‘measles’ and ‘respiratory tuberculosis’ to Chapter I: ‘Certain infectious and parasitic diseases’; ‘myocarditis’ to Chapter IX: ‘Diseases of the circulatory system’; and ‘pleurisy’, which can be a symptom of many conditions, to Chapter XVIII: ‘Symptoms, signs and abnormal clinical and laboratory findings, not elsewhere classified’. We then graphed the annual number of deaths occurring in each of the chapters from 1855–1949 and checked for sudden ‘breaks in slope’, where the number of deaths rose or dropped particularly dramatically. If the change in level persisted over a number of years, this alerted us to consider whether certain causes would need to move between chapters in order to create continuity over time. As a result, we reunited some causes, which, if diagnosed today, would be classed as ‘ill-defined’ or symptomatic, with more clearly diagnosed causes occurring in the same area of the body. For example, we placed ‘pleurisy’ together with ‘diseases of the respiratory system’ and ‘syncope’ with ‘circulatory diseases’. We merged some chapters which contained only small numbers and separated out some which were particularly important to the story of mortality in the nineteenth century: diarrhoea (including cholera, dysentery, enteric fever, enteritis, gastro-enteritis and typhoid, but excluding other diseases of the digestive system related to the liver, pancreas and intestines), tuberculosis (all forms) and old age (including ‘age’, ‘old age’, ‘senile decay’, ‘senile dementia’ and ‘senility’). This resulted in a classification scheme made up of a basic 15 categories, or ‘groups’, with which to consider mortality in Scotland. We then also applied this categorisation to the individual causes of death recorded in the death registers of Skye and Kilmarnock.^4^

## 3. Overview of mortality rates in Scotland

The work reported in this article sought to develop and extend the findings outlined in a previous article by the same authors (Reid, Garrett, Williamson, & Dibben, in press), which presented the raw numbers of deaths and both crude and standardised death rates by cause for Scotland from 1855 to 1949. The earlier article indicated that although there was relatively little change in the number of deaths overall in Scotland over the 10 decades we are studying, this must be set in the context of a rapid increase in population, despite considerable outmigration in certain periods, so that crude death rates in general were falling over most of this period. In addition, and particularly during the second quarter of the twentieth century, the age structure of the Scottish population changed as fertility fell, pushing a greater proportion of the population into the older ages, where mortality decline had not yet started to take hold. Age-standardised mortality rates therefore showed larger and more consistent falls as they progressed into the twentieth century than did crude mortality rates. As age- and sex-standardised mortality rates reflect the real risks of mortality within a population, mortality rates in the figures below are presented in age-standardised or age-specific forms.

**Figure 1:**
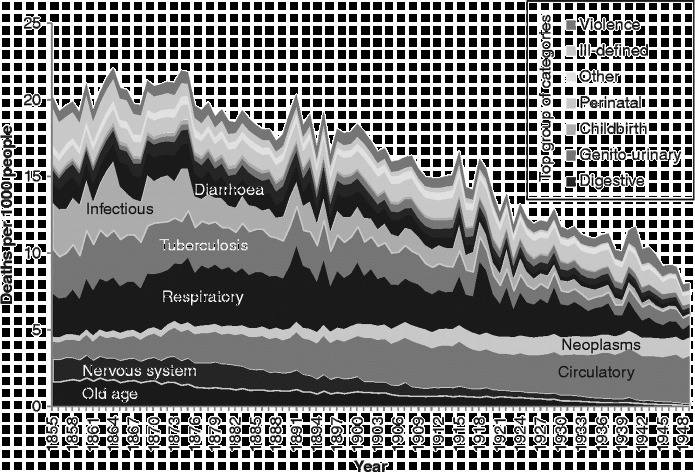
Age- and sex-standardised cause-specific mortality rates, Scotland, 1855–1949. Source: Davenport (2012).

Figure 1 shows annual age- and sex-standardised mortality rates for Scotland for the 15 broad causal groups designed to eliminate the discontinuities caused by classification and coding changes which we detailed above.^5^ The figure shows the familiar pattern of decreases in mortality rates from infectious and respiratory diseases, and increases in ‘degenerative diseases’ (circulatory diseases and cancers), described by Omran (1971) in his theory of epidemiological transition and forming the basis of McKeown's (1976a) analysis of the reasons for mortality decline.

However, it is also clear, from the way we have grouped our deaths and the fact that we have retained a specific category for ‘old age’ rather than placing this in the ‘ill-defined’ category as is common practice, that the increase in the risk of death from ‘degenerative diseases’, which are represented in our scheme by ‘circulatory disease’ and ‘neoplasms’ (cancers), is completely offset by the decline in the risk of death from old age and diseases of the ‘nervous system’. This prompted us to ask whether this pattern indicated that there had simply been a ‘rebranding’ of deaths due to ‘old age’. All the aforementioned diseases are most common amongst the elderly, and so, to explore this question further, we undertook an examination of causes of death by age.

**Figure 2:**
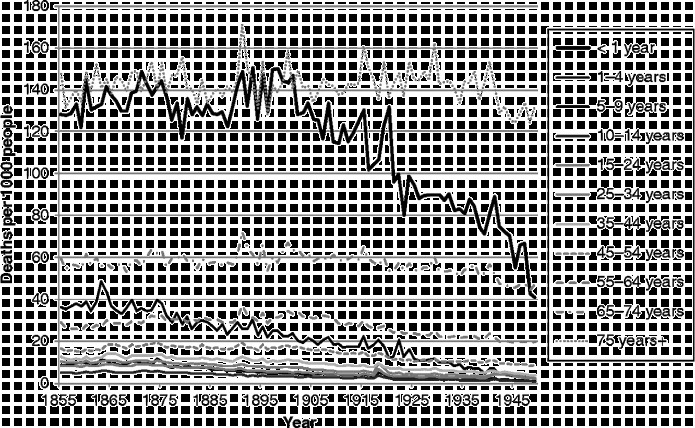
Age-specific mortality rates, Scotland, 1855–1949. Source: Davenport (2012).

First of all, it is worth reminding ourselves of the very different trajectory of mortality rates for different ages over time. Figure 2 shows age-specific mortality rates from all causes over the 1855–1949 period. The darker the lines in the figure, the younger the age group represented. In view of the graphs which follow, it is important to be aware not only of the trajectories over time in the risk of death at different ages, but also of the very different levels of those risks. The most vulnerable age groups throughout the period were the very young and the very old. Yet, while mortality amongst the very young declined rapidly from the beginning of the twentieth century, that amongst the very old did not start to decline until the very end of our period. Mortality decline started earliest, from the 1870s, amongst those in early childhood, with declines in later childhood and then early adulthood following.

**Figure 3:**
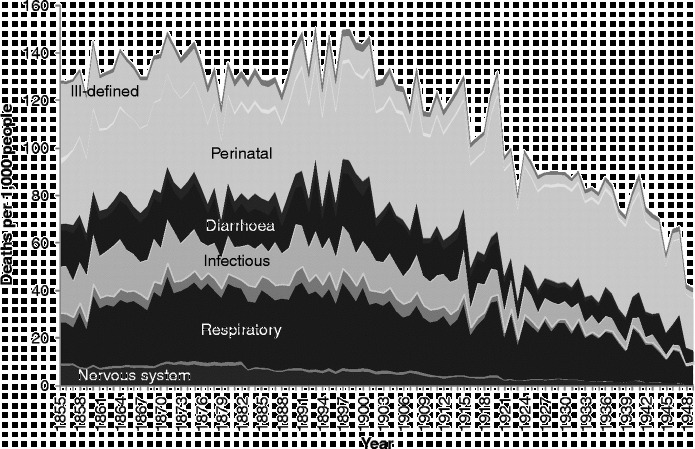
Cause-specific mortality rates for infants aged under 1 year, Scotland, 1855–1949. Source: Davenport (2012). Note: See Figure 1 for the detailed sequence of causes of death.

There is insufficient space here to show cause-specific graphs for all age groups, so we have selected some particular age groups as exemplars. Figures 3 4 5 6 7 show mortality rates by cause for the whole of Scotland for infants (those aged less than a year), those aged 1–4, men aged 25–34, women aged 25–34, and those aged 55 years and over, respectively. It is important to note that the vertical axis in each of these graphs is shown at a different scale to enable the composition of deaths in the lower mortality age groups to be examined more closely.

**Figure 4:**
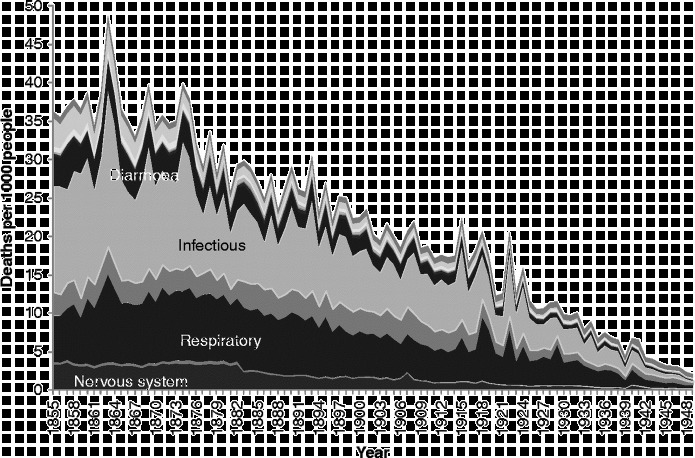
Cause-specific mortality rates for those aged 1–4 years, Scotland, 1855–1949. Source: Davenport (2012). Note: See Figure 1 for the detailed sequence of causes of death.

**Figure 5:**
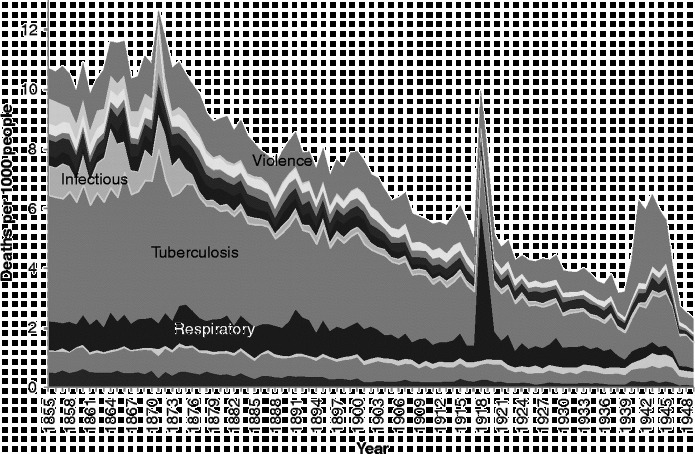
Cause-specific mortality rates for men aged 25–34, Scotland, 1855–1949. Source: Davenport (2012). Note: See Figure 1 for the detailed sequence of causes of death.

**Figure 6:**
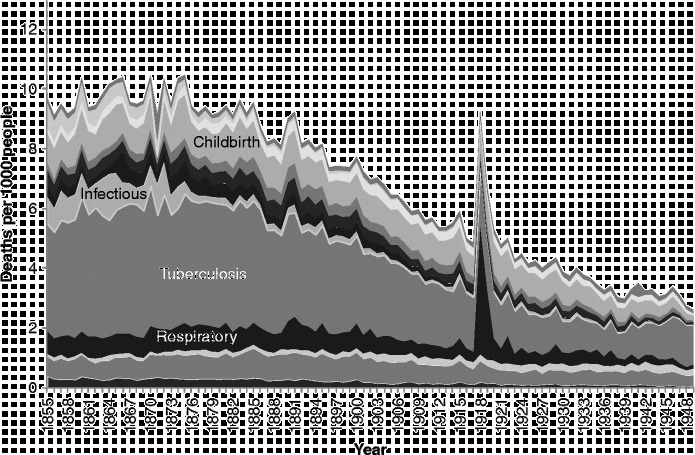
Cause-specific mortality rates for women aged 25–34, Scotland, 1855–1949. Source: Davenport (2012). Note: See Figure 1 for the detailed sequence of causes of death.

**Figure 7:**
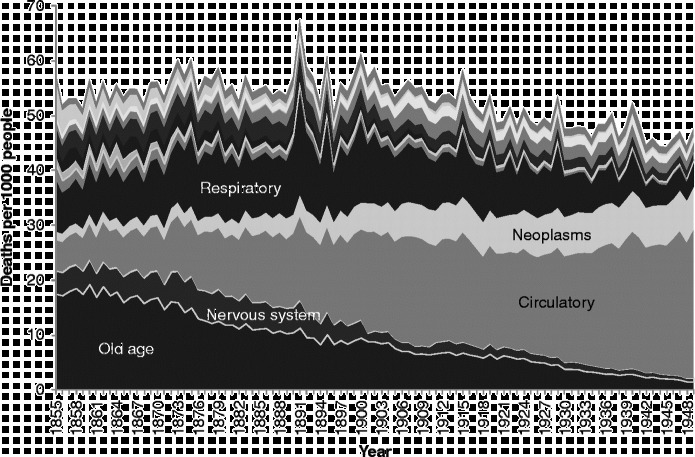
Cause-specific mortality rates for those aged 55 years and over, Scotland, 1855–1949. Source: Davenport (2012). Note: See Figure 1 for the detailed sequence of causes of death.

Figure 3 shows infant mortality rates. Throughout the period, a large proportion of infant deaths were due to the category we have termed ‘perinatal’, which includes causes such as prematurity, debility from birth, birth injury and congenital malformation. Infants in the nineteenth century were also vulnerable to death from infectious and respiratory diseases, as well as ill-defined causes. The series of long, hot summers in the 1890s, which led to high rates of diarrhoeal mortality and which preceded the decline in infant mortality, is clearly visible, as is the 1918 flu pandemic, which shows up both in mortality due to influenza, which falls in our ‘respiratory’ disease category, and as a peak in perinatal causes. It is likely that influenza in late pregnancy triggered early labour, resulting in weak and vulnerable infants, many of whom would have been unable to benefit from their mother's breast milk if she remained ill or had died herself (Reid, 2005). The bulk of the decline in mortality amongst infants in the first half of the twentieth century was in post-neonatal mortality and due to falls in exogenous causes such as infectious diseases and diarrhoea. Just visible in the graph, however, in the years immediately after the end of the Second World War, is the start of a major fall in neonatal mortality from perinatal causes.

Figure 4 shows that early child mortality (age 1–4) was dominated by infectious and respiratory diseases, and that these causes declined steadily throughout most of the study period, from the late 1870s onwards.

Mortality rates for adult men (Figure 5) were much lower than those for infants and children, and show a very different balance of causes. Infectious and respiratory diseases are still visible, demonstrating the very unusual characteristic of the 1918 flu pandemic, in that its main victims were young adults; usually influenza carries off the weakest members of society. Outbreaks of scarlet fever, diphtheria, smallpox and various types of diarrhoeal disease still attacked and killed a considerable proportion of adults in the third quarter of the nineteenth century. Violence, too, took a good proportion of young men throughout the study period, although mostly as a result of workplace accidents rather than other forms of violence; suicide was a small component within this category and homicide an even more minor one. Tuberculosis was the main killer of young men in Scotland across our study period, although deaths ascribed to this cause declined in tandem with infectious and respiratory diseases from the 1870s. It is thought that housing, hygiene and sanitary measures at a local level affected the incidence of and mortality from this infectious disease, although this is still debated (Davenport & Merler, 2013; Hardy, 1993, pp. 211–266; Woods, 2000, pp. 332–340).

It is important to note that the deaths shown in Figure 5 are those occurring amongst the civilian population. Nevertheless, the Second World War shows up as a significant mortality bulge formed from a wide variety of causes. This is likely to be a selection effect: those young men left at home contained a disproportionate number who were not fit enough to fight and were therefore at a much higher risk of death.

Deaths to adult women in the 25–34 age group (Figure 6) were also dominated by tuberculosis, but the decline in this cause for women, at least in Scotland, appears to have been delayed for a decade after it began amongst men. The 1918 flu pandemic affected women too, and throughout the study period a significant number of Scottish women also died of causes related to pregnancy and childbirth.

Amongst people in the ‘older’ age groups (Figure 7), which we have taken to be those aged 55 years and over, mortality rates were higher than amongst younger people, and the balance of causes was different yet again to that seen amongst those dying at younger ages. During the years from 1855–1949, infectious diseases (a pale band two above that showing deaths in the respiratory group) formed a very small proportion of deaths amongst the elderly, but the rate of deaths from these causes was very similar to the rate amongst younger adults, and, again, diarrhoeal diseases were prominent. Mortality rates from infectious and respiratory diseases in this age group fell over the period covered, but not dramatically, and other forms of mortality remained high, when the changes in scale between the graphs are taken into account. There is only a small increase in mortality with the 1918 influenza epidemic, but the increase in 1891 shows influenza in its usual form, affecting the vulnerable elderly population. It is interesting that mortality from old age and circulatory disease also appears to have been higher in the latter year. The greatest changes in the cause-of-death profile of the elderly occurred within the four groups at the bottom of the graph presented in Figure 7. In 1855, the largest of these ‘non-communicable’ causes was ‘old age’, sometimes referred to as ‘senility’. Along with deaths from causes related to the ‘nervous system’, deaths from ‘old age’ had declined almost to vanishing point by 1949, and ‘circulatory diseases’, which included heart disease and stroke, had come to dominate the ‘non-communicable’ group along with neoplasms. It is clear when the graphs of the various age groups are compared that the growth in cardiovascular diseases and cancers visible in Figure 1 was the result of the growth in these causes amongst the elderly. By showing ‘old age’ as a separate group in Figure 7, when it is usually assigned – and lost – amongst the ‘ill-defined’ causes, we highlight the fact that the growth in the risk of death from cardiovascular diseases was not a real growth but a rebranding of deaths from ‘old age’.

Today, the deaths of very few people are attributed to ‘old age’. There are plenty of people who die *in* old age from causes which are predominantly suffered by the elderly, but doctors seek to find and report the particular pathological cause which killed each person. In the past, such investigation was either not possible or not considered necessary. Deaths which today an autopsy would identify as having been due to cardiovascular disease would in the past simply have been attributed to old age. We wish to argue, therefore, that it might be misleading to talk about a *rise* in the risk of death from degenerative diseases. Of course, with the decline in infectious and respiratory diseases there is undoubtedly a shift in the balance of causes towards degenerative diseases, but this can happen without an increase in the risk of death from them: in 1855, a Scot aged 55 or over had about a 3 in 100 chance of dying from one of our four ‘non-communicable’ categories, and by 1949 this risk had scarcely altered. The chance that such deaths were ascribed to ‘circulatory disease’ rather than ‘old age’ or ‘diseases of the nervous system’ had quadrupled, but we argue that the risk of circulatory disease remained roughly constant.

## 4. Old age: doctors and lay informants

In our previous article, we were able to explore the phenomenon of deaths in old age in greater detail, and we demonstrated, using the information on the individual death certificates from Kilmarnock and Skye for the years between 1861 and 1901, that increasing levels of medical certification of cause of death are likely to have been a key factor in the decline in deaths from ‘old age’ (Reid et al., in press). Doctors were much more likely to have described the death of an elderly person as due to a cardiovascular or respiratory cause; it was lay informants – relatives or neighbours – who offered ‘old age’ as a cause of death. Greater medical certification, together with improvements in diagnosis, is likely to have contributed to the ‘rebranding’ of deaths.

Table 1 summarises the situation in our two communities and shows that, whether they were on the remote Isle of Skye or in the relatively cosmopolitan lowland town of Kilmarnock, medical men were much less likely than lay informants to attribute the death of an elderly person to old age. However, on Skye, a trend away from ‘old age’ as a cause of death is produced entirely by the increase in medical certification of cause of death, which rose from just over 12% to nearly 50% of all deaths to those aged over 55 between the 1860s and the 1890s – a trend mirrored by other insular rural districts in Scotland (Reid et al., in press). There was little change between the 1860s and the 1890s in the propensity of either doctors or lay informants on the island to ascribe elderly deaths to ‘old age’, but the number of parochial medical officers, and hence doctors, on Skye at any one time approximately doubled, increasing the possibility that a doctor would be available to certify a death. In Kilmarnock, however, not only was there a continued increase in the medical certification of cause of death (already at 90% in the 1860s – perhaps slightly ahead of Scotland's other large towns), but both doctors and lay informants attributed progressively fewer deaths among the elderly to ‘old age’. It is possible that improved diagnosis and medical fashion encouraged this trend amongst doctors in Kilmarnock, whose proximity to the medical school in Glasgow might have enabled them to be more in touch with medical trends than those on Skye: similar proportions of doctors in both places claimed membership of a medical society, but frequent attendance at meetings will have been impossible on Skye, and interaction between local doctors to spread new ideas will have been easier in Kilmarnock. However, we must also bear in mind the possibility that any tendency away from using ‘old age’ as a cause of death amongst doctors on Skye might be rendered invisible by an increase in certification without a corresponding increase in medical care before death. Even if a doctor certified a cause of death, he may only have seen the deceased in the final moments of their life, or indeed not until after death, and therefore may have been heavily reliant on the reports of relatives to assign the cause of death. Much of the increase in medical certification on Skye could have been comprised of such incidences, which afforded the doctor little knowledge of the cause of death, even if he had wished to assign it to a more aetiologically meaningful cause. A similar social, rather than aetiological, argument may explain the decrease in deaths attributed to old age by lay informants in Kilmarnock. If patients were increasingly willing to visit or be seen by a doctor in their last illness, and thereby acquire a diagnosis of what was ailing them, more relations who were unable to have the death certificate signed by a doctor may nevertheless have felt able to supply a more precise cause when registering the death.

**Table 1:** Deaths aged 55 years and over by medical or lay certification and whether or not attributed to ‘old age’.

	1860s	1870s	1880s	1890s
Number of deaths aged 55 and over				
Kilmarnock				
Not medically certified	129	68	27	10
Medically certified	1159	1284	1420	1663
Skye				
Not medically certified	1164	1046	967	874
Medically certified	167	435	593	768
Percentage of deaths aged 55 and over attributed to ‘old age’				
Kilmarnock				
Not medically certified	69.77	64.71	66.67	50.00
Medically certified	12.86	13.24	7.25	5.65
Skye				
Not medically certified	31.19	25.14	37.75	38.33
Medically certified	13.77	8.28	8.60	15.10
Percentage of deaths aged 55 and over medically certified				
Kilmarnock	89.98	94.97	98.13	99.40
Skye	12.55	29.37	38.01	46.77

Source: Death registers of Skye and Kilmarnock (1861–1901).

## 5. Multiple causes of death

One influence on the change in reporting is a change in the amount of detail provided by doctors, and lay informants, for inclusion on the death certificates. Both the published results and our analysis of the individuals in the death registers attribute each death to just one underlying cause, even if more causes were written on the certificate or in the register. The propensity to give more than one cause of death and how multiple causes were allocated to a single underlying cause provide another layer of complexity to our understanding of trends in causes of death.

The pro forma death certificate drawn up under the 1855 Registration Act for Scotland (An Act to provide for the better, 1854) for completion by doctors had only a single space for cause of death. There is no similar pro forma provided in the Acts for England and Wales, but certificates in use under the Births and Deaths Registration Act 1874 and submitted as evidence to the Enquiry into the Registration of Deaths of 1893 (Select Committee on Death Certification, 1893, p. 251) indicate that they included spaces for both primary and secondary causes. The enquiry also covered Scotland and Ireland, and an Irish certificate is provided in the report (Select Committee on Death Certification, 1893, p. 263), showing just a single space, but there is no Scottish certificate. It is doubtful, however, whether the provision of spaces for both primary and secondary causes actually aided the accuracy of cause of death, as several witnesses to the enquiry testified to the ‘confusion in the minds of medical men as to the meaning to be attached to the words ‘primary’ and ‘secondary’ … the words are interpreted by some as meaning ‘the primary cause chronologically, and by others as the primary cause physically of death’’ (Select Committee on Death Certification, 1893, p. xvii). Recommendations made as a result of the enquiry appear to have been put into effect because, by 1911, a circular regarding certificates of causes of death included a footnote to the effect

that acute specific diseases, if of recent occurrence, are to be considered the primary cause of death, even though the actual disease, as tested by power of infection, be no longer present at the time of death, eg measles (primary), five weeks; bronchopneumonia (secondary) ten days. (General Register Office, 1911)

**Figure 8:**
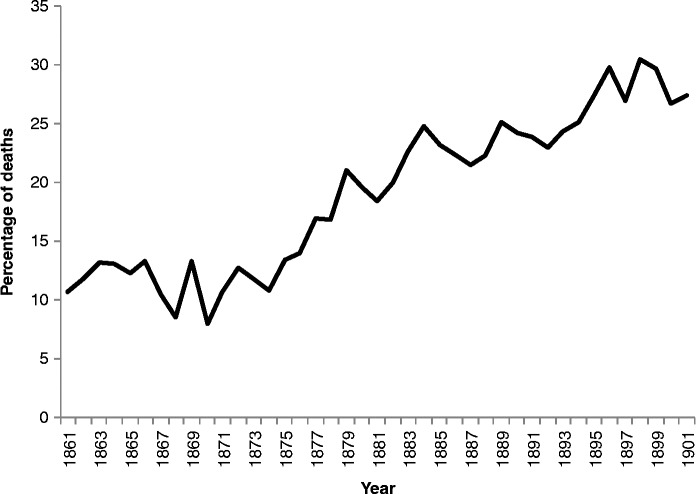
Percentage of deaths in Skye and Kilmarnock (combined) where more than one cause was offered. Source: Death registers of Skye and Kilmarnock (1861–1901).

So, although it is not clear whether the Scottish death certificates included separate spaces for primary and secondary causes, it is clear that, increasingly, doctors were offering up more than one cause. Inspection of the individual registrations for Skye and Kilmarnock shows phrases such as ‘acute pneumonia following measles’ and ‘epilepsy followed by apoplexy’ throughout the whole period for which data are available. Assuming that the registrars copied the cause of death faithfully from the certificate into the register, these phrases suggest both that only one space was available and that the notions of primary and secondary causes did not influence the way in which these were written down. Figure 8 shows that there was a considerable increase in the proportion of deaths where more than one cause was recorded in our two Scottish communities over the second half of the nineteenth century.

Just as we have been unable to find instructions to doctors on how to fill in and order causes on a certificate, we have also been unable to find instructions to clerks on how to allocate causes to one primary or underlying cause for inclusion in the statistical tables.^6^ It is possible that they took the first recorded cause, but they may have taken the last recorded cause or considered all causes recorded in the register entry and made an assessment based on medical knowledge, a set of rules or a combination of the two. It is also possible that different clerks used different decision-making processes. Although there are no instructions available, we can try to establish whether any procedures were being followed by clerks by comparing the individual registrations with the published deaths by cause for Kilmarnock.

**Figure 9:**
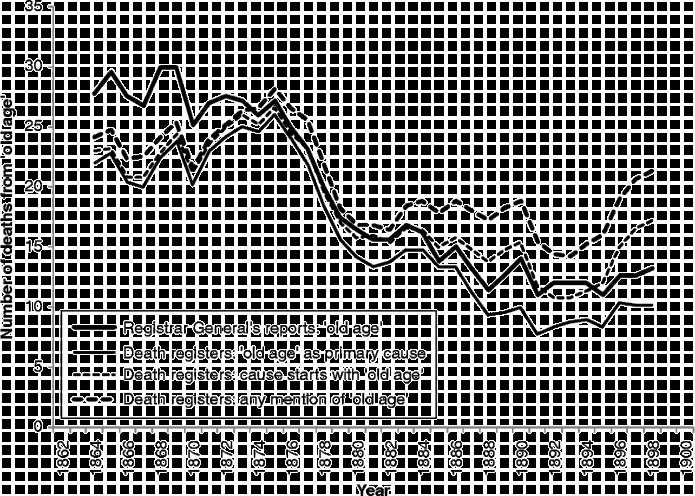
Number of deaths from ‘old age’ in Kilmarnock, 1862–1900, comparing the Registrar General's reports and death registers (five-year moving averages). Sources: Registrar General's detailed annual reports and death registers of Kilmarnock (1861–1901).

The thick solid line in Figure 9 shows the numbers of deaths attributed to ‘old age’ in Kilmarnock in the Registrar General's detailed annual reports. The figure also shows the numbers derived from the individual death registrations, according to three different treatments: the thin solid line shows our assessment of the numbers of deaths where ‘old age’ was the primary cause if modern-day coding rules are followed. Today's coding rules clearly state that a death must be attributed to the temporally antecedent condition which could have started a chain of other causes, but that, wherever possible, deaths should not be attributed to ill-defined and uninformative causes. Thus, ‘old age’ should not be chosen as the primary cause of death where a more precise cause of death is also offered. For example, if the cause of death was simply ‘old age’ or ‘senility’, there is no choice: we have attributed the death to old age. If, however, more than one cause is recorded in the register, an assessment is needed. If ‘infirmities and old age’ was recorded, we also attributed the death to ‘old age’, as ‘infirmities’ is an even less informative cause of death than ‘old age’, but if ‘chronic bronchitis and old age’ or ‘senile debility/congestion of lungs’ is written, the death is attributed to the more precise cause.

The numbers of deaths we have attributed to old age are universally smaller than those attributed by the Registrar General's clerks. It is possible that they were simply taking the first-mentioned cause on a death certificate, or maybe they were attributing any death which mentioned ‘old age’ to that general category. The two dashed lines show that neither of these possibilities provides a good fit. It appears that, in the 1860s and early 1870s, the registrars were attributing a significant number of additional deaths to old age, over and above those which mentioned old age, senility, senile dementia or a similar cause on the death certificate. During the early 1870s, a fundamental shift took place, which affected both the propensity for doctors to attribute deaths to ‘old age’ and the coding practices of the clerks, who started to attribute fewer deaths to ‘old age’ than mentioned on the registration forms, although the numbers were still greater than we would have attributed using modern-day coding rules. The coincidence of the change in recording practices amongst doctors with that in classification amongst the clerks suggests that there might have been an edict from higher up in the Registrar General's office, but unfortunately we can find no trace of such a proclamation in the published reports or General Register Office letter books.^7^

It is clear, however, that in the early 1870s the Registrar General for Scotland appears to have undergone a sea change in his attitude towards ‘old age’. It is important to note that these reports and the statistics behind them were written and prepared not by the Registrar General himself, but by his Superintendent of Statistics. Until 1874, this post was held by Dr James Stark but, due to a backlog in the preparation of reports, the 1870 report was the last one he prepared (Scottish Way of Birth and Death, n.d.). Those from 1871 to 1878 were written by Dr William Robertson, who appears to have had a very different view of the value of ‘old age’ as a cause of death. In the detailed annual report for 1870, Stark noted the number of deaths from ‘Old Age without marked disease’ with no further comment (General Register Office, 1874, p. xl), although a few pages later he also wrote:

it is one of the peculiarities of the Scottish race, that they use every effort to ascertain the exact age of the deceased, so that, as a general rule, the age at death may be relied upon as being correct. (p. xli)^8^

The following year, although Robertson also reported the number of deaths from ‘old age’ without comment (General Register Office, 1875b, p. xliv), he maintained that ‘the age attributed to a person at death … is not much to be depended on’ (pp. xliv–xlv). By the 1872 report, he was condemning the use of ‘old age’ as a cause of death, as well as deriding the accuracy of ages provided among the old: ‘It is quite plain that the term Old Age, when used as denoting a cause of death, is little better than a confession of ignorance, and greatly vitiates our statistics, especially in Insular Rural districts’ (General Register Office, 1876, p. xlvi). He made similar comments in the following years, and the coincidence of his official appointment in 1874 with the change in both the recording and classification of deaths from ‘old age’ in Kilmarnock suggests that, although we have not yet found any record of this, he somehow communicated his views to at least some doctors who were in the business of certifying deaths.^9^

## 6. Individual doctors' practices

The individual death registrations for Kilmarnock also contain the name of the doctor who certified the death. Examination of the way in which individual doctors certified deaths amongst the elderly can shed light on the way in which Dr Robertson's realisation that ‘old age’ was an uninformative cause of death filtered through into practice, and the process of change. There were between 10 and 20 doctors practising every year in Kilmarnock, although, due to population growth, the number of doctors per 10,000 people stayed roughly constant at about six. Inevitably, there was a reasonable degree of turnover amongst these medical men, as older doctors retired or died and were replaced by younger doctors. Many of these younger men stayed for only a brief portion of their career, using their stay in Kilmarnock to gain experience, but others settled in the town for many years.

**Figure 10:**
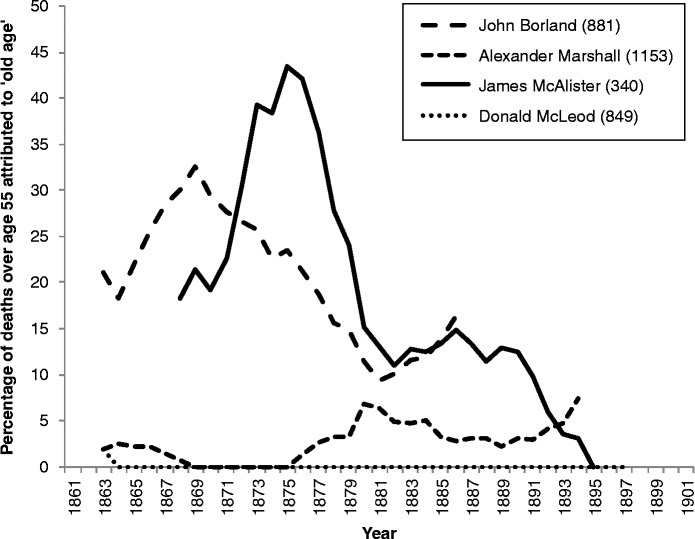
Deaths due to ‘old age’ amongst individual doctors in Kilmarnock, 1861–1901. Source: Death registers of Kilmarnock. Note: Five-year moving averages are shown. The numbers in parentheses are the total numbers of deaths aged 55 and over certified by each doctor.

In order to gain robust enough numbers to examine trends in the causes of death amongst the elderly, we have restricted our analysis to the four doctors who each certified over 300 deaths for those aged 55 and over during their careers in Kilmarnock. All four were practising in Kilmarnock from the early 1860s until the late 1880s or 1890s. Figure 10 shows the proportions of deaths of the elderly which they attributed to ‘old age’ and, as with Figure 9, five-year moving averages are shown.

It is clear from Figure 10 that these four individual doctors had very different attitudes to cause of death amongst the elderly. John Borland and James McAlister appear to reflect the aggregate pattern, reducing their use of ‘old age’ as a cause of death over the 1870s, with Borland acting promptly or even anticipating the Superintendent of Statistics' views. It is likely that there was a more general discussion within medical circles which prompted other doctors, as well as Dr Robertson, to adopt new thinking on the usefulness of ‘old age’ as a cause of death. Indeed, the president's 1863 address in the Public Health Department of the Social Science Association, delivered by a Scottish doctor in Edinburgh, had used the same term – ‘confession of ignorance’ – as Dr Robertson, when describing deaths from atrophy, sudden death, teething and old age (Christison, 1863, p. 438). James McAlister's reduction in the use of ‘old age’ as a cause was delayed for a few years and followed an apparent reaction against the general trend, although the smaller numbers of deaths certified by McAlister means that less weight should be placed on the exact sequence of his trend. Perhaps more interesting is the fact that the remaining two doctors shown in Figure 10 were never in the habit of attributing deaths amongst the elderly to ‘old age’, despite certifying a considerable number of such deaths across their many years of established practice in Kilmarnock. Indeed, Donald McLeod only certified one death using ‘old age’ without adding a more informative cause of death.

It seems that while some doctors reacted to a shift in attitude towards ‘old age’ as a cause of death, others were ahead of their time. This does not appear to have been linked to their cohort or training school: all four doctors included in Figure 10 trained in Glasgow, and those more keen to attribute deaths to ‘old age’ were both the youngest and the oldest of the four. It is possible that it is linked to the age or social profile of the doctors' patients, but the average age of the four doctors' patients within the 55 and over age group remained constant at around 70 years of age over the period depicted in the graph. It is noticeable that the patients whose deaths were classed as being from ‘old age’ were significantly older. The patients who Dr McAlister certified as dying from this cause were aged 77 on average; those of Dr Borland were 80; and those of Dr Marshall 85. In the case of Dr McAlister, his shift in certification during the 1870s was not linked to an increasing concentration of ‘old age’ amongst his most elderly patients – there was no discernible change over time in the average age of those certified by him as dying from old age. The average age of death of Dr Borland's patients who died from ‘old age’ did creep up (while that of all his patients dying aged 55 years and over did not), but there was no discernible break in the 1870s to accompany his reduction in use of this cause of death. It appears that both Dr Borland and Dr McAlister started to look for alternative causes amongst all patients over the age of 55, not just those at the younger end of the elderly category.

## 7. Old age and the shift to other causes of death

**Figure 11:**
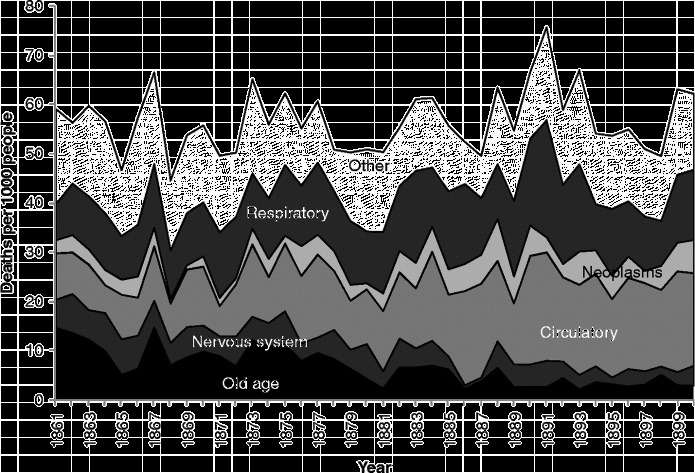
Cause-specific mortality rates for those aged 55 and over, Kilmarnock, 1861–1900. Source: Death registers of Kilmarnock (1861–1900).

We speculated above that the shift away from ‘old age’ as a cause of death was due to the rebranding of deaths amongst the elderly as other causes, particularly ‘circulatory diseases’. Figure 11 shows an equivalent graph to Figure 7 for Kilmarnock, based on the individual causes of death. Readers should bear in mind that Figure 11 only goes up to 1900, so they should not expect the right-hand side of the graphs to look the same. The numbers are smaller, so there are considerably more fluctuations, and, for simplicity, only the four ‘degenerative’ categories and respiratory diseases are shown separately, the rest of the groups being amalgamated into one ‘other’ category. As in Figure 7, we can see a general rise in respiratory diseases over the 1870s and 1880s, and it is important to consider the possibility that ‘old age’ deaths were being transferred into ‘respiratory diseases’ rather than ‘circulatory diseases’ and ‘neoplasms’.

**Figure 12:**
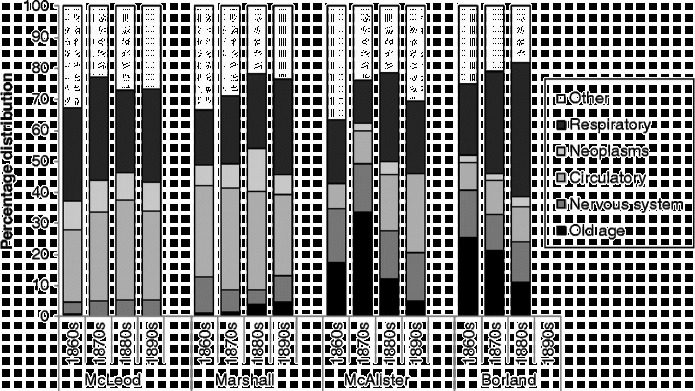
Distribution of causes of death amongst those aged 55 and over by decade for four doctors, Kilmarnock, 1861–1901. Source: Death registers of Kilmarnock (1861–1901).

We can examine this possibility by looking at how the balance of causes of death amongst the elderly changed amongst the four selected doctors in Kilmarnock. Figure 12 shows the distribution of causes of death amongst those aged 55 and over for each doctor, across the four decades between 1861 and 1901. The two doctors whose certification patterns changed away from ‘old age’ show markedly different patterns. Dr McAlister's decline in ‘old age’ deaths was mainly made up for in ‘circulatory’ deaths, but also deaths from ‘respiratory’ diseases, but that of Dr Borland was compensated for almost entirely by the latter. It seems that any instruction to move away from the use of ‘old age’ did not discourage doctors from having ‘favourite’ causes of death – they simply shifted to a different favourite. However, respiratory disease was probably an easy cause to light upon if searching for a more aetiologically meaningful cause of death. While bronchitis might be thought of as an easily recognisable condition, we should, however, ask whether this is any more likely to have reflected an underlying cause of death than, say, ‘paralysis’ or ‘old age’. Respiratory conditions are frequently a complication accompanying or following another disease, and the fact that some doctors were keener than others to use this category suggests that respiratory disease should be regarded as a symptom which other doctors might have identified as having a different underlying cause. Hardy (1993, p. 229) suggests that tuberculosis deaths were sometimes recorded as respiratory diseases, and it is also possible that deaths from respiratory complications of infectious diseases such as measles or whooping cough were attributed to pneumonia or bronchitis.

However, it is possible that respiratory diseases were more severe in the latter years of the nineteenth century amongst the elderly, and the fact that Dr Marshall's deaths in this age group (very few of which were attributed to old age) shifted somewhat towards the respiratory category supports this. However, Dr McLeod showed no increasing tendency to identify respiratory deaths amongst the elderly, suggesting that there might not have been a real increase and that Dr Marshall was increasingly using ‘respiratory’ in preference to other causes, just as Dr Borland was using it in preference to ‘old age’.

It is worth noting that Drs Borland and McAlister, who favoured ‘old age’ as a cause of death, also had a fondness for ‘diseases of the nervous system’, which in the 55 and over age group were mainly seen on the certificates as the rather uninformative term ‘paralysis’. Both Drs McLeod and Marshall, on the other hand, certified more deaths as being due to ‘neoplasms’ and ‘circulatory disease’. It would seem that they may have subscribed to a different philosophy of mortality causation from that of Drs Borland and McAlister.

It is also worth pointing out that the four doctors varied in their propensity to use multiple causes of death. Drs Marshall and McLeod did not even list ‘old age’ in conjunction with another cause – when the latter was mentioned at all, it was as a sole cause. Similarly, Dr Borland used ‘old age’ as a single cause or not at all, so when he made his abrupt move away from certifying ‘old age’ as a cause of death, he did not mention the latter on the death certificates he subsequently completed. Dr McAlister, however, as well as being later in his move away from ‘old age’, carried on mentioning it on as many death certificates as before, but he started to add another cause: commonly, ‘chronic bronchitis’, ‘heart failure’ or ‘paralysis’. This doctor was clearly more reluctant than Dr Borland to change his established mode of behaviour.

## 8. Reconciling the local and the national

Returning to national patterns, the dramatic shift in the propensity to attribute deaths to ‘old age’ seen in Kilmarnock is not clearly visible in the national figures. However, the contrast between Skye and Kilmarnock and between the different doctors in Kilmarnock has shown that a strong temporal influence can be severely muted, and its effect on the overall pattern attenuated, by different responses amongst individuals and by the balance of various individuals, or groups of individuals, in different places.

We have seen that some doctors in Kilmarnock, who perhaps valued precision in their practice of medicine, were ahead of their time in their approach to ‘old age’ as a cause of death, while others were at the vanguard of a change in attitude. This is perhaps not surprising, given Kilmarnock's precocity in terms of medical certification of death. Kilmarnock was a thriving place, with a good middle-class clientele, as well as interesting work in the hospital and the availability of additional income for medical men in the form of insurance work and retainers for surgeons who were prepared to offer their services to particular firms or industrial sectors. The town's transport connections and proximity to Glasgow are likely to have made it an attractive place for doctors to want to practice, and, once there, it would have been easy for them to keep up with medical controversy and debate, should they have so wished.

Skye was at the other extreme, with a very high level of uncertified deaths and little apparent change in doctors' practices. It is unfortunate that the low number of medically certified deaths and the very high turnover of doctors on the island prevents examination of the causes individual doctors were entering on the death certificates. Most other places in Scotland will have fallen somewhere in between these two different scenarios, and the resulting aggregation is likely to have produced the impression of a gradual change away from the use of ‘old age’ as a cause of death.

## 9. Conclusions

We began this article by considering two of the ‘grand theories’ of historical demography: Omran's theory of epidemiological transition and the ‘McKeown thesis’ (Omran, 1971, McKeown 1976a). This led us, like many previous researchers, to examine changes in cause of death over time, and then to explore a number of issues relating to how causes of death were assigned by those reporting them and by those compiling volumes of national statistics. Initially, we examined a broad range of causes of death over time and at different ages, culled from the national statistics for Scotland, but we came to focus on deaths amongst the elderly population, which we defined as those aged 55 years and over, using the individual death certificates drawn from two contrasting Scottish communities. In a previous article (Reid, Garrett, Williamson and Dibben, in press), we were able to consider how lay people and doctors differed in the causes of death they reported, and here we examined the causes of death which certain individual doctors chose to enter on a death certificate, and how this differed over time. By comparing our observations at the local level with the published statistics for Kilmarnock, we have been able to extrapolate between the two in order to gain a clearer picture of the ‘black box’ whereby individual causes were assigned to particular cause-of-death categories by the clerks in the Registrar General's office. We were even able to gain a better understanding of how the clerks dealt with multiple causes on one certificate, and to consider some of the implications of the increasing tendency to report such multiple causes, as medical certification of cause of death became increasingly sophisticated.

One unexpected outcome of our research was the opportunity to trace the way in which gains in knowledge, perhaps made by just a few significant people, can filter down through the population to change the behaviour of a significant proportion of the overall population. Nosological changes occur abruptly, from one year to the next, as a new classification scheme is brought in by the Registrar General to replace one thought to be outdated. The change may have been discussed over many years before being implemented, but it often appears as a dramatic discontinuity, or break, in the time series of causes of death. Changes in the recording of deaths by doctors do not happen as suddenly and cannot be detected through discontinuities. The foibles of a group of doctors in a particular community, and the make-up of the group, can influence just how quickly a new ‘preferred cause’ might be adopted. This process, replicated over many communities, may make it difficult to put a precise chronology on when changes in registration protocol came about, and just when changing medical knowledge, or fashion, began to alter the cause-of-death profile of a particular community, or indeed of Scotland as a whole.

The existing literature on cause-of-death reporting has, we have argued, neglected ‘old age’ as a category of cause of death. By extracting deaths due to ‘old age’ from the class of ‘ill-defined’ causes to which they have long been relegated, and examining cause-specific mortality amongst those aged 55 years or more, we have raised the possibility that the rise in mortality rates due to cardiovascular diseases and cancers, which played such a prominent role in Omran's epidemiological transition theory, was not due to a rise in these ‘degenerative’ diseases, but was the result of improving diagnosis. We argue that the apparent rise in the risk of death from cardiovascular diseases and cancers observed in so many European populations in the late nineteenth and early twentieth centuries is not real, but an artefact caused by progressively fewer deaths from such diseases being attributed to ‘old age’ and ‘paralysis’. Our detailed analysis was only able to look at the last 40 years of the nineteenth century, although we had figures at the national level which extended until 1949, and these strongly suggest that there was very little increase in the risk of dying from a degenerative disease over the late nineteenth century and the first half of the twentieth century. Of course, a shift in the balance of causes did occur, with degenerative diseases, including ‘old age’, forming an increasing proportion of all deaths, and this is the essence of the epidemiological transition. However, it is important to realise that this does not necessarily mean that rates went up in absolute terms. The epidemiological transition theory is accepted with virtually no criticism (although Mackenbach (1994) argues that it is poorly-defined). We do not wish to deny its validity, but rather want to warn against its overenthusiastic interpretation. We therefore wish to argue that demographers, and others, need to refrain from using the theory, as it has been too often used, to suggest that the risk of degenerative diseases increased within the population of any particular age group. Infectious diseases were certainly failing to carry off the population at younger ages, but people who escaped death in childhood or early adulthood and survived into old age in the early twentieth century faced approximately the same risk of death from the same sort of degenerative diseases and conditions as the elderly in previous generations. Later in the twentieth century, of course, the risk of succumbing to death from such diseases became increasingly delayed.

We have also argued that changes in certification, registration and coding practices may well have affected the numbers of deaths attributed to respiratory disease. The fact that the cause profiles returned by different doctors in the same place and with the same sort of clientele varied so widely (some never used the term ‘old age’, while others seem to have swapped to other ‘symptomatic’ causes) reinforces the caution needed when interpreting nineteenth-century causes of death. Particular caution should be exercised when comparing cause-of-death data for different places, as it is shown here that diagnostic patterns varied greatly from place to place, and medical knowledge and recording practices changed at different speeds in different places. Comparing the same place over time might be less problematic if the same doctors remained active in that place for several decades, for it is likely that they maintained their own diagnostic preferences over time, although clearly some did respond to changing medical fashion.

The shift to respiratory disease detected amongst the elderly should caution researchers against accepting at face value respiratory disease deaths reported for other ages, particularly as this is the one set of causes which is prominent as a killer throughout the life course. Because respiratory disease is often a complication of an infectious disease or a non-communicable condition, which are the true underlying cause of death, doubts about the validity of a respiratory cause cast doubt on the reliability of trends – even those relating to more ‘easily identifiable’ causes. If trends in respiratory deaths and other broad categories of cause of death do, indeed, have a wide margin of error, such that the shift in the balance between respiratory diseases and other fatal conditions cannot be interpreted with confidence, this has important implications for McKeown's argument that rising standards of living *per se* were sufficient to bring about the decline in mortality seen over our study period (McKeown, 1976a); for Szreter's contradictory public health thesis (Szreter, 1988); for Woods and Shelton's comparisons of groups of causes by time and place (Woods and Shelton, 1997); and, indeed, for any attempts to relate mortality decline to specific interventions by analysing trends in cause-specific mortality.

All the points we have raised would merit further detailed analysis, using individual death certificates where possible to counterpoint the body of published statistics, but we feel that the implications for the work of McKeown and Omran, on which so much other research has been based, need to be teased out as a matter of priority.

## Footnotes

^1.^ See also McKeown (1976b); McKeown and Brown (1955); McKeown, Brown, and Record (1972); McKeown and Record (1962); and McKeown, Record, and Turner (1975).

^2.^ For more recent summaries of the debates, see Colgrove (2002) and Grundy (2005).

^3.^ For more detail on the certification of death in Scotland and on differences between Scotland and England and Wales, see Crowther (2006) and Davis (2007).

^4.^ Our approach therefore differs to those of scholars such as Wolleswinkel-Van den Bosch et al. (1996) and Meslé and Vallin (1996), who try to follow individual detailed causes of death over time, aggregating up to maintain aetiological consistency and correctness. In contrast, we start with broad cause-of-death groupings of interest and transfer individual causes to maintain smooth trends.

^5.^ In order to calculate standardised rates, annual age, sex and cause-specific mortality rates were applied to the 1891 population to generate the expected number of deaths in the 1891 population.

^6.^ Inspection of the open letter books in the National Archives of Scotland (General Register Office for Scotland, 1870, 1875a, 1880) suggests that the General Register Office was more concerned with making sure that cause of death was recorded and, if possible, certified than with the precise way this was done.

^7.^ This does not mean that there was no edict. The letter books (General Register Office for Scotland, 1870, 1875) contain correspondence with individual registrars, and the volume on ‘correspondence containing registration matters, received and issued by the RG’s [Registrar General's] office’ (General Register Office for Scotland, 1880) does not contain all the circulars referred to in other volumes.

^8.^ The General Registrar for Scotland produced both annual reports and detailed annual reports. The Registrar General for England and Wales produced just one much lengthier annual report.

^9.^ Coincidentally, in 1874, the Registrar General for England and Wales issued a new certificate for the certification of death, together with a complete nomenclature of causes (British Medical Journal, 1874). It is unclear whether this list of causes included ‘old age’ as an acceptable cause, as in 1842 (General Register Office, 1842, pp. 97, 110), or condemned it as an unauthorised, indefinite or otherwise undesirable term, as in 1911 (General Register Office, 1911). The main proponents of more precise diagnosis in both countries were certainly reading and referring to each other's work (Rumsey, 1867, p. 198), and it is possible that other Scottish doctors were also influenced by developments south of the border, and might have seen and taken to using any new list of causes produced.
